# Comparison of Routine Preoperative Ultrasound-Guided Airway Examination Versus Clinical Airway Examination in Predicting Difficult Laryngoscopy in Patients Coming for Elective Surgery: An Observational Study

**DOI:** 10.7759/cureus.73225

**Published:** 2024-11-07

**Authors:** Geront Andrews, Sriraam Kalingarayar, Amar Nandhakumar, Balaji PR, Balaji Kesavan, Nandeeswaran Kola Sridharan

**Affiliations:** 1 Anaesthesiology and Critical Care, University Hospital of North Tees, Stockton on Tees, GBR; 2 Anaesthesiology, Kovai Medical Center and Hospital, Coimbatore, IND; 3 Anesthesiology, Kovai Medical Center and Hospital, Coimbatore, IND; 4 Critical Care Medicine, Royal Victoria Infirmary and Freeman Hospital, Newcastle Upon Tyne, GBR; 5 Anaesthesiology, Meenakshi Mission Hospital & Research Centre, Madurai, IND

**Keywords:** cormack and lehane grade, difficult airway, difficult laryngoscopy, modified mallampati test, neck circumference, tongue volume

## Abstract

Background

As part of preoperative patient evaluations, it is customary to estimate the likelihood of difficulties during laryngoscopy and intubation. A diverse array of predictors is frequently employed by anesthesiologists to anticipate difficult laryngoscopy (DL); however, no single predictor has been established as the gold standard. In the present study, we compared routine preoperative ultrasound-guided airway examination and clinical airway examination in predicting DL in patients coming for elective surgery.

Methodology

The present study is a single-blinded observational study that spanned 12 months, beginning in March 2018 and concluding in February 2019. The study took place at Kovai Medical Center and Hospital (KMCH), which is a tertiary-care facility in Coimbatore, India. The data were obtained through face-to-face interviews, with a sample size of 135 research participants. The questionnaire contained comprehensive data regarding the patient’s age, weight, gender, comorbidities, planned surgery, and preoperative assessment, which encompassed a detailed examination of the airway. The clinical airway examination comprised assessing mouth opening, performing the modified Mallampati test, measuring thyromental height (TMH) and thyromental distance (TMD), evaluating neck circumference, and observing neck extension. Ultrasound-guided airway examination was performed during the immediate pre-operative period, focusing on specific parameters such as the pre-epiglottic space, anterior neck soft tissue at the level of vocal cords, thyrohyoid membrane, suprasternal notch, hyoid bone (single parameter measured at different levels), hyo-mental ratio, and tongue volume (TV).

Results

Among the ultrasound parameters, TV (72.15 cm^3^) is the best ultrasound parameter to predict DL (95% CI: 0.65 to 0.82), with a high sensitivity of 82% and specificity of 45.88%. Other parameters that are useful are anterior neck soft tissue at the level of the thyrohyoid membrane (0.67 cm), suprasternal notch (1.01 cm), and, lastly, pre-epiglottic space (0.57 cm). Among clinical parameters, modified Mallampati grade (grade 3 or more) is the best parameter to assess the airway clinically and predict DL, followed by neck circumference (>42 cm) and TMH (>5 cm). Based on the findings, we observed that both clinical and ultrasound parameters are comparable to predict DL.

Conclusion

We observed that routine clinical airway examination and ultrasound examination yielded comparable results in predicting DL. Therefore, the routine use of clinical airway examination still holds good in predicting DL.

## Introduction

Safe airway management is the primary concern of all anesthesiologists. The term "difficult airway" refers to a variety of medical scenarios, including incapacity or difficulty in performing intubation, laryngoscopy, or respiratory ventilation. Difficult face mask ventilation occurs when an anesthesiologist cannot maintain a patient's oxygen saturation above 92% or effectively manage ventilation despite using a positive-pressure mask during general anesthesia [1]. The modified Cormack-Lehane grade of the laryngeal view offers the most comprehensive description of the laryngeal inlet during direct laryngoscopy [2,3]. Difficult laryngoscopy (DL) is a term typically employed to describe the identification of a grade 2b, 3, or 4 view during the procedure [3,4]. The American Society of Anesthesiologists (ASA) Task Force states that intubation of the trachea must be attempted more than once before it can be considered challenging, regardless of whether the tracheal disease is present or not [5]. To prepare for airway management under general anesthesia or as a backup for regional anesthesia, it is necessary to conduct a preliminary examination of the airway. This enables us to anticipate possible intubation and laryngoscopy difficulties and to plan accordingly.

The prevalence of airway difficulty linked to each definition of DL exhibits variation. There are three parameters: poor view at laryngoscopy (grade 3-4) documented on modified diagrams of Cormack and Lehane, three or more laryngoscopy attempts, and failure of direct laryngoscopy. Among the three parameters, researchers found that 15.8% of patients with a grade 3-4 view required three or more laryngoscopies. Conversely, among those who underwent three or more laryngoscopies, 84.1% had a grade 3-4 view [6]. This suggests a higher probability of encountering challenging intubation in patients who experience DL. Studies suggest that DL occurs in the operating room (OR) at a prevalence rate of 2% to 16% [7]. Therefore, in the field of anesthesia, it is essential that one anticipates challenging laryngoscopy.

The widely used parameters to evaluate the airway clinically comprise mouth opening (MO), Mallampati grading [8] (later modified by Samsoon and Young) [9], thyromental distance (TMD), thyromental height (TMH) [10], neck circumference (NC), range of neck movements, and condition of teeth [11]. The clinical evaluation of the airway helps resolve potential complications; however, this assessment is confined to the oral cavity. Hence, an uncomplicated airway during clinical examination may present challenges during laryngoscopy or intubation, posing a significant risk to the patient’s life if not promptly addressed.

In recent times, ultrasound airway assessment has emerged as a rapidly growing and extensively researched subject. Its significance lies not only in predicting difficult airways during pre-operative assessments but also in managing airway assessments in intensive care units where clinical evaluations may be impractical. One disadvantage could be the significant learning curve associated with it. Parameters employed in earlier studies include anterior neck soft tissue at the vocal cord level, thyrohyoid membrane, suprasternal notch, anterior neck soft tissue at the hyoid level (15 mm to the right and left of the midline) [12], pre-epiglottic space (PES) [13], hyo-mental ratio (HMR), and tongue volume (TV) [14].

Considering the benefits and drawbacks associated with each modality and recognizing the scarcity of ultrasound use in preoperative airway evaluation, the employment of ultrasound-guided airway assessment is assumed to serve the purpose of predicting DL and identifying potentially valuable ultrasound parameters. Subsequently, ultrasound parameters are examined for correlation with clinical parameters to determine which one is a more reliable predictor of DL.

## Materials and methods

Study design, period, and participants

The present study is a single-blinded observational study that spanned 12 months, beginning in March 2018 and concluding in February 2019. The study took place at Kovai Medical Center and Hospital (KMCH), which is a tertiary-care facility in Coimbatore, India.

Inclusion criteria

Individuals who met the following criteria were considered for inclusion: 18-65 years of age, body mass index of 18-24 kg/m^2^, and the need for general anesthesia and intubation for elective surgical operations (ASA grades 1-2).

Exclusion criteria

We did not include patients who expressed a desire not to participate, those requiring immediate surgical attention, or those who presented with masses in the neck.

Sample size and sampling technique

To find out how useful point-of-care ultrasound is for evaluating challenging laryngoscopy, Adhikari et al. performed follow-up research. Anesthesiologists diagnosed challenging laryngoscopy in twelve percent of the participants [12]. In accordance with this prevalence rate (p), the sample necessary for this study was determined using the formula N = 3.84 * p * q / d2, where q represents the complement of p and d represents a 6% absolute error. It was determined that the investigation necessitated a minimum of 113 samples. All 135 participants who met the selection criteria have been incorporated.

Data collection procedure

Data were gathered from study participants via in-person interviews. The questionnaire comprised comprehensive details about the patient’s age, weight, gender, planned surgery, and preoperative assessment, which encompassed a detailed examination of the airway. To provide anxiolysis and prevent aspiration, all patients were given tablet alprazolam (0.5 mg) with tablet pantoprazole (40 mg), and tablet metoclopramide (10 mg) the evening before the surgical procedure.

Clinical parameters

Mouth Opening

The patients were instructed to sit in a chair with proper back support and requested to fully open their mouths. Subsequently, the distance between the upper and lower incisors was measured using a tape. Observers noted difficulty in laryngoscopy when the MO measures less than 4.5 cm [15].

Modified Mallampati Test

The patients were asked to sit up straight with their head held in neutral and to open their lips wide, stretching their tongue out as far as it would go, without phonating. The presence of modified Mallampati test (MMT) grades 3 and 4 suggests a DL [16].

Thyromental Distance

While sitting up straight, the patients were asked to maintain their jaw clenched and their neck and head extended to the maximum as they could. From the mentum's interior to the thyroid prominence's external surface, the observer measured a straight line. When the distance was equal to or is less than 6.0 cm, we expected DL [17,18]. We made the identical measurement while keeping the head in a neutral position.

Thyromental Height

Specifically, we measured the distance from the front edge of the thyroid cartilage, which is located on the thyroid predominance connecting both thyroid laminae, to the front edge of the mentum, which is on the mandible's mental protuberance. We took these measurements with the individual resting supine position and their lips sealed [19]. When the height was equal to or is less than 5.0 cm, we expected DL.

Neck Circumference

In both the standing and sitting positions, we measured NC at the middle point of the thyroid cartilage using a conventional tape measure [20]. We considered NC of 42 cm or greater as an indicator of DL.

Neck Extension

We instructed the patients to sit on a chair with proper support for their shoulders and spines and asked them to maintain a neutral position for their neck. The patient was requested to extend their neck, ensuring that their shoulders remained immobile. The angle was determined using a goniometer, which measured the plane from the external auditory canal to the tip of the nose [20]. We considered laryngoscopy to be difficult if the neck extension (NE) was below 35.

Teeth

From the population studies, those with buck teeth, those with missing teeth, and those partially or fully edentulous were considered difficult candidates for laryngoscopy.

In the immediate pre-operative period, an ultrasound-guided examination of the airway was conducted.

Ultrasound data recording

The patients were directed to lie in a supine posture. To measure different sonographic parameters, both the linear high-frequency probe (L14-5/38, operating at 14-5 MHz) and the curvilinear low-frequency probe (C5-2/60, operating at 7-3 MHz) of the ultrasound machine (GE LOGIQ-e BT12) were used. To capture transverse measurements along with mid-sagittal, the probes were systematically placed beneath the participant’s chin at various points. To measure certain anatomical features, the transverse view was employed, encompassing the width of the tongue, PES, the anterior neck soft tissue at the level of vocal cords (NTVC), thyrohyoid membrane, and suprasternal notch. The cross-sectional area of the tongue and the mento-hyoid distance were measured using the mid-sagittal view, specifically in neutral and hyper-extended positions. We evaluated the soft tissue thickness at the level of the hyoid bone using parasagittal views, measuring 15 mm to the right and left of the midline. Before starting the study, the operator under supervision of a trained radiologist conducted 50 ultrasound examinations and data collection, following which 20 individual examinations were performed, which achieved a Kappa agreement of greater than 0.8 with the supervisor.

Laryngoscopy

Upon entering the OR, pre-induction monitors such as oxygen saturation (SpO2), non-invasive blood pressure, and ECG were connected via the Philips MP50-IntelliVue multi-parameter monitor. An 18 gauge intravenous cannula was properly secured, and an intravenous fluid infusion was started. The induction of general anesthesia involved the administration of intravenous fentanyl at a dosage range of 1-2 mcg/kg, followed immediately by propofol at a dosage range of 1-2 mg/kg after a 20-second interval. The patients received assisted ventilation using 100% oxygen alongside isoflurane. Muscle relaxation was achieved by administering atracurium intravenously at the specified dose, followed by a flush with IV fluid lasting 5-10 seconds. The train-of-four (TOF) measurement with the Organon TOF watch neuromuscular monitor (MIPM Mammendorfer Institut für Physik und Medizin GmbH, Mammendorf, Germany) was used to assess the achievement of neuromuscular blockade.

The duration between the administration of the neuromuscular relaxant and the point at which the TOF drops below 20% was regarded as the onset time of neuromuscular blockade. Once adequate pre-oxygenation was achieved and stable vitals were confirmed, a consultant anesthesiologist conducted laryngoscopy to assess the difficulty level using the modified Cormack-Lehane grading. This classification system assigns a grade, ranging from grade I to grade IV [2], as given in Table 1. The Cormack-Lehane classification system categorizes laryngeal views into two groups: grades I and IIa, which indicate easy visualization of the larynx (EVL) and suggest straightforward intubation, and grades IIb, III, and IV, which represent difficult visualization of the larynx (DVL) and typically indicate potential intubation challenges [21].

**Table 1:** The Cormack–Lehane grading The Cormack–Lehane grading system is a way to describe the view of the larynx during laryngoscopy [2].

Grade	Structures visualized
I	Entire laryngeal aperture
IIa	Posterior portion of the laryngeal aperture with partial view of the glottis
IIb	Posterior portion of the laryngeal aperture (arytenoids or posterior vocal cords only)
III	Only epiglottis
IV	No visualization of the larynx or epiglottis

Ethical considerations

We received approval from the institutional ethics committee of KMCH Limited, Coimbatore, before the start of our study (approval number: EC/AP/566/10/2017). The participants were individually given a comprehensive explanation, and before the collection of data, their signed consent was obtained from the principal investigator.

Data analysis

All the data were imported into Microsoft Excel (Microsoft Corp., Redmond, WA), and the analysis was performed using SPSS Version 26.0 (IBM Corp., Armonk, NY). The receiver operating characteristic (ROC) curve was used to establish difficult values and to estimate specificity, sensitivity, confidence interval (CI), and Cohen's kappa coefficient of agreement for the purpose of evaluating agreement. For expressing continuous variables, mean and standard deviation were used. For expressing the categorical data, frequency and percentages were used. The optimal cutoff values for ultrasound parameters were determined using Youden's index, which was calculated from the ROC curves [22].

## Results

A total of 135 individuals, of whom 80 were male (59.3%) and 55 were female (40.7%), were examined. The mean age of patients was 45.74 years. Table 2 presents the basic characteristics of the research participants.

**Table 2:** Basic attributes of the research participants

Parameter	Male (n=80)	Female (n=55)	Minimum	Maximum
	Mean ± SD	Mean ± SD		
Age	44.71 ± 12.74	47.25 ± 12.02	18	60
Weight	67.98 ± 8.19	65.09 ± 8.30	49	103
Height	1.67 ± 0.07	1.58 ± 0.06	1.45	1.83
BMI	25.94 ± 3.41	24.32 ± 3.26	18.21	39.24

In this study, 82 (60.8%) patients belonged to ASA class I, while 53 (39.2%) patients belonged to class II. Table 3 shows the gender-wise distribution of the ASA physical status of the study population.

**Table 3:** Gender-wise distribution of ASA physical status of the research participants ASA, American Society of Anesthesiologists

ASA Classification	Male	Female	Total
ASA I	49	33	82
ASA II	31	22	53
Total	80	55	135

Table 4 shows the Cormack-Lehane grading of study participants. Overall, 45 (33.4%) patients were under grade IIb and 40 (29.6%) patients were under grade III. The percentage of patients who had DL, as measured by a Cormack-Lehane grade of 2b or above, was 62% in our research.

**Table 4:** Cormack-Lehane grading of study participants. Cormack-Lehane classification is a four-grade system describing laryngeal view during direct laryngoscopy

Cormack-Lehane Grade	Frequency	Percentage
I	11	8.1
IIa	39	28.9
IIb	45	33.4
III	40	29.6
IV	0	0.0
Total	135	100

ROC curves were used to define difficulty values and find the specificity, sensitivity, CI, and Cohen’s kappa coefficient of agreement for the purpose of evaluating agreement.

Ultrasound parameters

**Figure 1:**
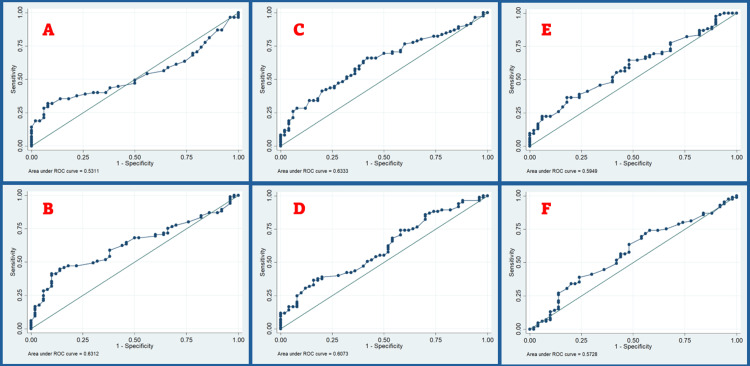
ROC curve for (A) neck soft tissue at vocal cords, (B) neck soft tissue at the thyrohyoid membrane, (C) neck soft tissue at suprasternal notch, (D) neck soft tissue at the hyoid 15 mm to the right of the midline, (E) neck soft tissue at the hyoid 15 mm to the left of the midline, and (F) pre-epiglottic space. This figure shows six distinct ROC curves for various neck soft tissue measurements and pre-epiglottic space, serving as predictors for DL (A-F). Neck soft tissue measurements at the thyrohyoid membrane, suprasternal notch, and pre-epiglottic space are the better predictors. DL, difficult laryngoscopy; ROC, receiver operating characteristic

Figure 1 shows the ROC curve for neck soft tissue at vocal cords, thyrohyoid membrane, suprasternal notch, hyoid bone, and PES. In this study, the defined cut-off value for NTVC, as determined by the ROC curve, was 0.33 cm. This value exhibited a sensitivity of 49.4% and a specificity of 50%. Based on this parameter, we predicted that 67 patients, which accounted for 50.37% of the population, would have a DL. The level of agreement between NTVC and laryngoscopy was determined to be 49.63%. The calculated area under the curve (AUC) was 0.53 (95% CI: 0.43 to 0.63), which did not reach clinical significance (p = 0.53).

As per the ROC curve, the defined cut-off value for neck soft tissue at the thyrohyoid membrane (NTTM) was 0.67 cm, with a sensitivity of 68.2% and a specificity of 48%. We made the prediction that 83 (61.48%) patients would face difficulties during laryngoscopy, and there was a 61.48% agreement between NTTM and laryngoscopy. The calculated AUC was determined to be 0.63, with a 95% CI ranging from 0.54 to 0.72, showing statistical significance (p=0.02).

As per the ROC curve, the cut-off value for neck soft tissue at the suprasternal notch (NTSN) was defined as 1.01 cm, showing a sensitivity of 65.8% and a specificity of 58%. We made the prediction of DL for 77 (57.04%) patients, with a 62.96% agreement rate between NTSN and laryngoscopy. The calculated AUC was 0.63 (95% CI: 0.54 to 0.74), showing statistically significant (p=0.003).

As per the ROC curve analysis, the established cut-off value for the neck soft tissue at hyoid 15 mm to the right of the midline (NSTR) was 1.02 cm. A sensitivity of 54.1% and a specificity of 54% were observed at this value. Based on this parameter, it was determined that 69 (51.11%) patients exhibited challenging laryngoscopy. The agreement level between NSTR and laryngoscopy was found to be 54.07%. The observed AUC was 0.61 (95% CI: 0.51 to 0.7), showing no statistical significance (p=0.18).

As per the ROC curve analysis, the established cut-off value for the neck soft tissue at hyoid 15 mm to the left of the midline (NSTL) was 0.94 cm. A sensitivity of 64.7% and a specificity of 52% were observed at this value. Based on this parameter, it was determined that 79 (58.52%) patients exhibited challenging laryngoscopy. The agreement level between NSTL and laryngoscopy was found to be 60%. The observed AUC was 0.59 (95% CI: 0.5 to 0.69), showing no statistical significance (p = 0.18).

As per the ROC curve, the established cut-off value for the PES was 0.57 cm. The observed value showed a sensitivity of 63.5% and a specificity of 52%. Based on this parameter, we predicted that 78 patients, which accounted for 57.78% of the population, would have a difficult airway. It was found that there is a 59.26% level of agreement between PES and laryngoscopy. The AUC, with a calculated value of 0.57 (95% CI: 0.47 to 0.67), demonstrated clinical significance (p = 0.04).

**Figure 2:**
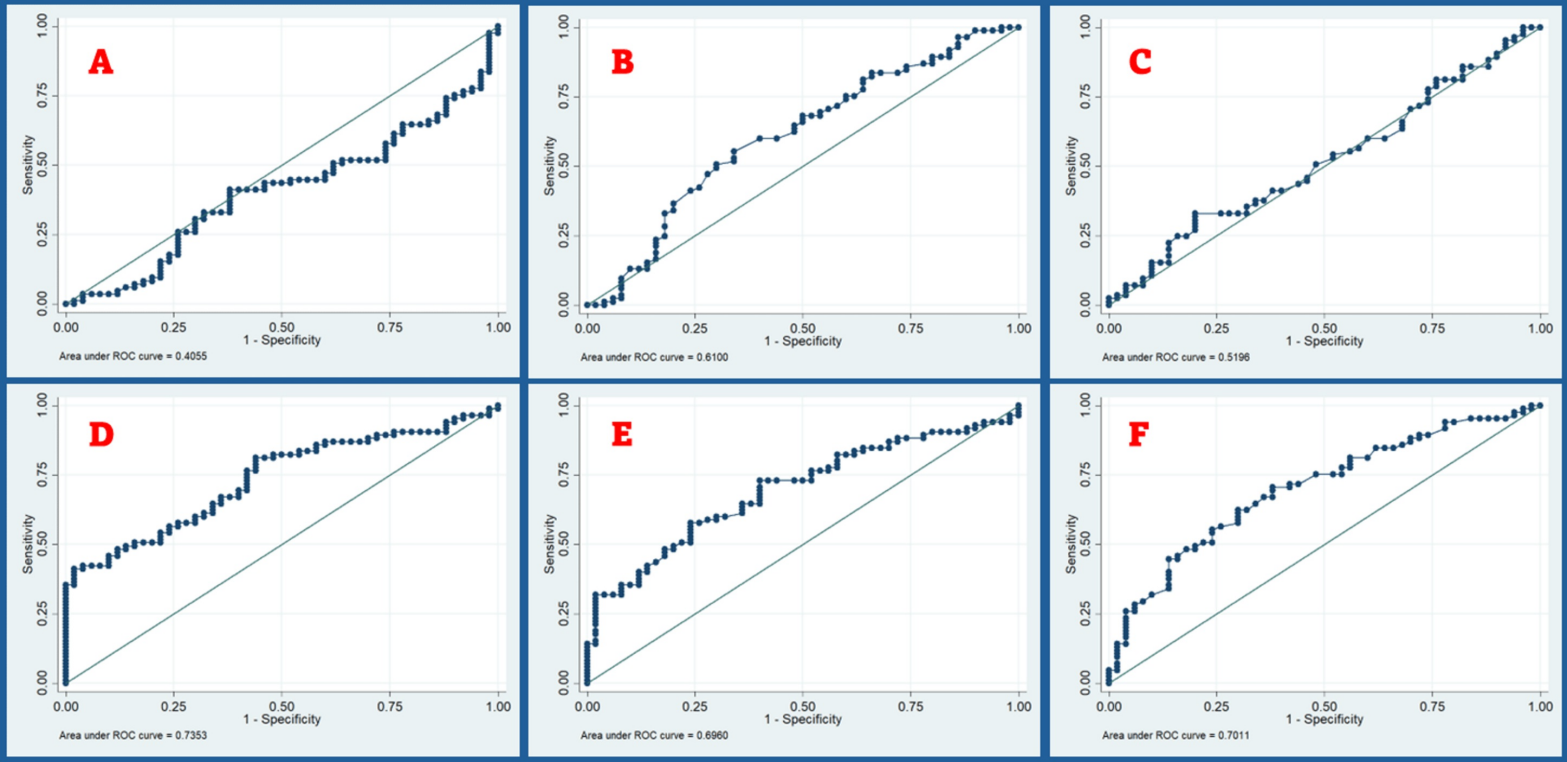
ROC curve for (A) hyo-mental ratio, (B) hyo-mental distance in neutral position, (C) hyo-mental distance in hyper-extended position, (D) tongue volume, (E) tongue area, and (F) tongue width. The figure shows six distinct ROC curves for various hyoid and tongue measurements, serving as predictors for DL (A-F). Tongue measurements (volume, area, width) are better predictors than hyoid measurements, and hence tongue measurements should be given more weight in airway assessment. DL, difficult laryngoscopy; ROC, receiver operating characteristic

Figure 2 shows the ROC curve for HMR, hyo-mental distance in neutral and hyper-extended positions, and TV along with area and width of the tongue. The calculated AUC for the HMR by ROC was determined to be less than 0.5 (95% CI: 0.3 to 0.5), leading to the conclusion that the test was deemed unhelpful, and no further analysis was conducted.

The distance from the hyoid to the mentum in neutral position (HMD-N), as defined by the ROC curve, was found to be 4.78 cm. This value displayed a sensitivity of 68.2% and a specificity of 50%. The number of patients predicted to have a challenging laryngoscopy was 83 (61.48%), with a corresponding agreement rate of 61.48%. The determined AUC was found to be 0.61 (95% CI: 0.51 to 0.71), signifying statistical significance (p = 0.02). The distance from the hyoid to the mentum in hyper-extended position (HMD-HE), as defined by the ROC curve, yielded a value of 5.98 cm, with a sensitivity of 50.59% and a specificity of 52%. The assessment revealed that 57 (49.63%) patients were expected to face challenges during laryngoscopy, with an agreement rate of 51.11%. The statistical analysis revealed an AUC of 0.52 (95% CI: 0.42 to 0.62), which did not reach significance (p=0.39).

The calculated AUC for TV by ROC was determined to be 0.74 (95% CI: 0.65 to 0.82), leading to the conclusion that this test is the most useful among ultrasound parameters for detecting difficult airways. In terms of DL, the ROC analysis established a value of 72.15 cm^3^, with a specificity of 82% and a sensitivity of 52%. The level of agreement between TV and laryngoscopy was determined to be statistically significant (p = 0.001) at a rate of 70.37%.

The ROC curve analysis revealed that the cut-off value for the tongue area (TV-A) was determined to be 22.47 cm^2^. The sensitivity and specificity observed at this value were 72.94% and 60%, respectively. Based on this parameter, it was determined that 82 (60.74%) patients exhibited challenging laryngoscopy. The level of agreement between the tongue area and laryngoscopy was determined to be 68.15%. The observed AUC was 0.70 (95% CI: 0.61 to 0.78), showing statistical significance (p=0.000).

As per the ROC curve analysis, the cut-off value for the width of the tongue (TV-T) was determined to be 3.45 cm. At this value, the sensitivity and specificity were observed to be 71% and 58%, respectively. An agreement rate of 66.67% was determined between the tongue area and laryngoscopy. The AUC, as observed, was 0.70 (95% CI: 0.61 to 0.78), signifying statistical significance (p=0.000). Table 5 shows the sensitivity and specificity rates of ultrasound parameters.

**Table 5:** Ultrasound parameters NTVC, neck soft tissue at vocal cords; NTTM, neck soft tissue at the thyrohyoid membrane; NTSN, neck soft tissue at the suprasternal notch; NSTR, neck soft tissue at hyoid 15 mm to the right of the midline; NSTL, neck soft tissue at hyoid 15 mm to the left of the midline; PES, pre-epiglottic space; HMD-N, hyo-mental distance in neutral position; HMD-HE, hyo-mental distance in hyper-extended position; HMR, hyo-mental ratio; TV-A, tongue area; TV-T, width of the tongue; TV, tongue volume

Parameter	Defined Value	AUC	Sensitivity	Specificity	p-Value
NTVC	0.33	0.53	49.4	50	0.53
NTTM	0.63	0.63	68.2	48	0.02
NTSN	1.01	0.63	65.8	58	0.00
NSTR	1.02	0.61	54.1	54	0.18
NSTL	0.94	0.59	64.7	52	0.03
PES	0.57	0.57	63.5	52	0.04
HMD-N	4.78	0.6	68.2	50	0.02
HMD-HE	5.98	0.5	50.59	52	0.39
HMR	-	<0.5	-	-	-
TV-A	22.47	0.69	72.94	60	0.00
TV-T	3.45	0.7	71	58	0.00
TV	72.14	0.73	82	52	0.00

Clinical parameters

**Figure 3:**
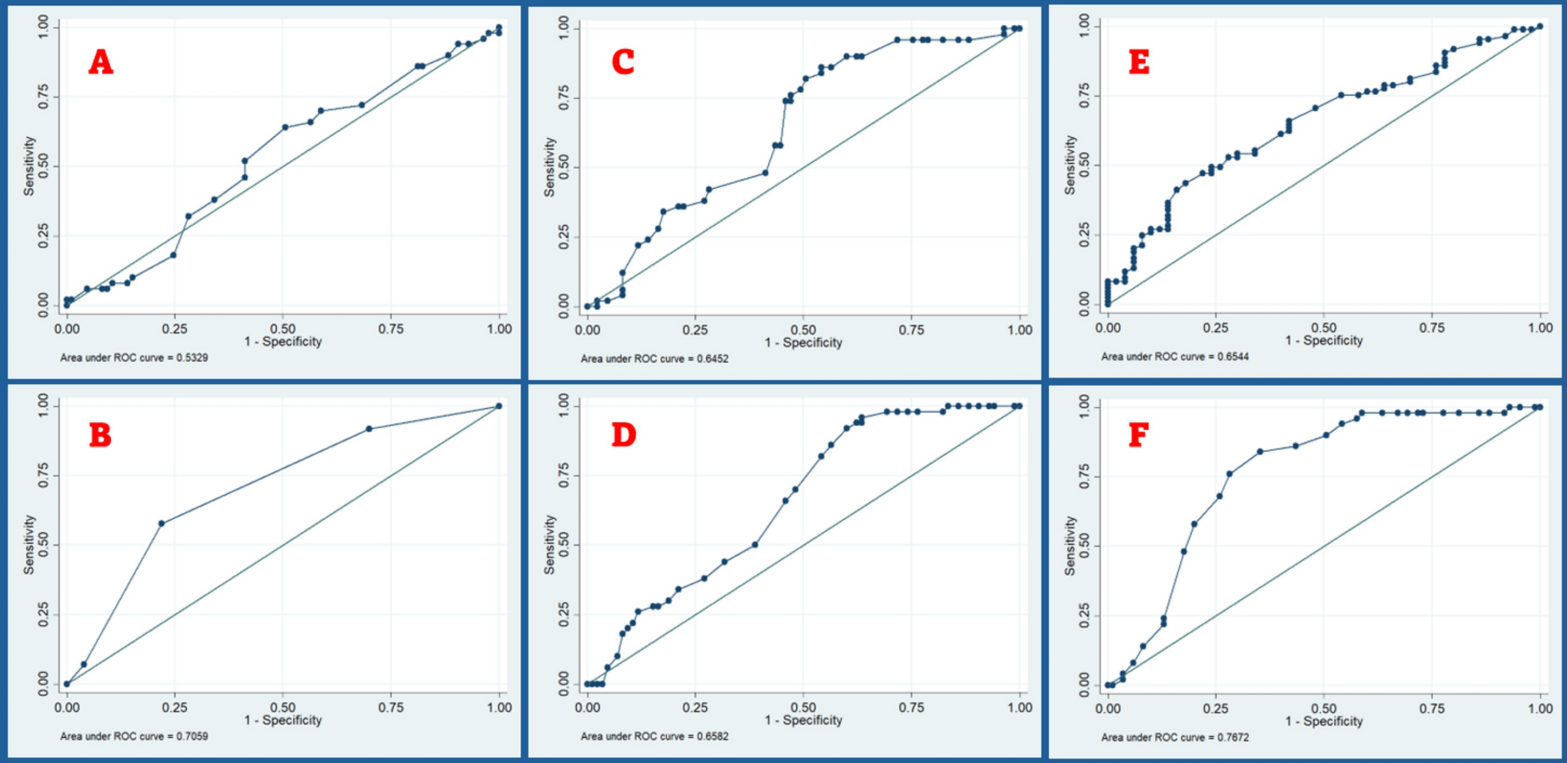
ROC curve for (A) mouth opening, (B) modified Mallampati test, (C) thyromental distance, (D) thyromental height, (E) neck circumference, and (F) neck extension. The figure shows six different ROC (receiver operating characteristic) curves (A-F). Except for mouth opening, all the other five clinical parameters appear to be reliable predictors of DL. DL, difficult laryngoscopy; ROC, receiver operating characteristic

Figure 3 shows the ROC curve for MO, MMT, TMD, TMH, NC, and NE. The ROC curve analysis showed a cut-off value of 3.8 cm for MO. At this specific value, the observed sensitivity and specificity were 98% and 2.35%, respectively. The agreement level between the MO and laryngoscopy was determined to be 37.78%. The AUC was observed to be 0.53, with a 95% CI of 0.43 to 0.63, showing no statistical significance (p = 0.45).

The MMT indicated a sensitivity of 57.65% and a specificity of 78% for a grade of 3 or higher. Based on this parameter, it was determined that 60 (60.74%) patients exhibited challenging laryngoscopy. A 65.19% level of agreement was observed between the Mallampati grade and laryngoscopy. The AUC, as observed, yielded a value of 0.71 (95% CI: 0.62-0.79), signifying statistical significance (p = 0.00).

If the TMD exceeds 5.9 cm, it is deemed difficult for laryngoscopy. The ROC curve for TMD showed an AUC of 0.65 (95% CI: 0.55 to 0.74). According to this study, the sensitivity at a distance of 5.9 cm was 100%, while the specificity was 1.18%. The level of agreement between TMD and laryngoscopy was determined to be 39.26%, which reached statistical significance (p=0.00).

If the TMH exceeds 4.9 cm, it is deemed difficult for laryngoscopy. The ROC curve for MO showed an AUC of 0.66 (95% CI: 0.57 to 0.75). According to this study, the sensitivity at a distance of 4.9 cm was 82%, while the specificity was 45.88%. The level of agreement between TMH and laryngoscopy was determined to be 58.52%, which reached statistical significance (p = 0.01).

Laryngoscopy becomes difficult when the NC surpasses 42 cm. The AUC for the ROC analysis of NC is 0.65, with a 95% CI of 0.56 to 0.75. According to this study, the sensitivity at a distance of 42 cm was 47.06%, while the specificity was 78%. The level of agreement between NC and laryngoscopy was determined to be 58.52%, which reached statistical significance (p = 0.00).

Laryngoscopy becomes difficult when the NE falls below 35º. The ROC curve analysis for NE indicates an AUC of 0.77, with a 95% CI ranging from 0.69 to 0.85. The study reported a sensitivity of 100% and a specificity of 1.18% at 35º. The level of agreement between NE and laryngoscopy was determined to be 37.78%, which did not reach statistical significance (p = 0.22). The agreement reached for teeth was 49.63%, which was deemed statistically significant. The agreement reached for teeth was 49.63%, which was deemed statistically significant. Table 6 shows the sensitivity and specificity rate of clinical parameters.

**Table 6:** Clinical parameters MO, mouth opening; MMT, modified Mallampati test; TMD, thyromental distance; TMH, thyromental height; NC, neck circumference; NE, neck extension

Parameter	Gold Standard Values	AUC	Sensitivity	Specificity	p-Value
MO	3.8	0.53	98	2.35	0.45
MMT	3	0.71	57.65	78	0.00
TMD	5.9	0.65	100	3.53	0.08
TMH	4.9	0.65	82	45.88	0.01
Teeth	-	-	-	-	0.00
NC	42	0.65	47.06	78	0.00
NE	350	0.77	100	1.18	0.22

Comparison of clinical parameters and ultrasound parameters

The significant parameters from the clinical group and ultrasound group were analyzed individually. The combined sensitivity and specificity for predicting DL were analyzed separately for each group, with an increasing number of parameters. Table 7 shows the prediction of combined clinical parameters, with increasing order of difficulty.

**Table 7:** Prediction of combined clinical parameters with increasing order of difficulty. The above table reveals that with an increase in the number of parameters for a patient, sensitivity decreased and specificity increased. The specificity reached 100% with just three parameters. The use of Mallampati test, neck circumference, thyromental height, and teeth condition together yields a specificity of 100%. Even with the use of three or more of the above parameters, the specificity remained 100% in predicting difficult laryngoscopy.

Cut-off	Sensitivity	Specificity
≥0	100.00%	0.00%
≥1	87.00%	42.00%
≥2	60.00%	84.00%
≥3	25.88%	100.00%
≥4	3.53%	100.00%
>5	0.00%	100.00%

Table 8 shows the prediction of combined ultrasound parameters with increasing order of difficulty. In the above table, we can see that specificity increased but sensitivity decreased as the number of difficult patient parameters increased. The attainment of 100% specificity was only possible by employing all five parameters. According to the results, the combined assessment of NTTM, NTSN, PES, HMD-N, and TV shows a 100% specificity in predicting DL.

**Table 8:** Prediction of combined ultrasound parameters with increasing order of difficulty. The combined assessment of NTTM, NTSN, PES, HMD-N, and TV shows 100% specificity in predicting DL. NTTM, neck soft tissue at the thyrohyoid membrane; NTSN, neck soft tissue at the suprasternal notch; PES, pre-epiglottic space; HMD-N, hyo-mental distance in neutral position; TV, tongue volume; DL, difficult laryngoscopy

Cut-off	Sensitivity	Specificity
≥0	100.00%	0.00%
≥1	97.65%	8.00%
≥2	87.06%	30.00%
≥3	55.29%	76.00%
≥4	32.94%	88.00%
>5	0.00%	100.00%

**Figure 4:**
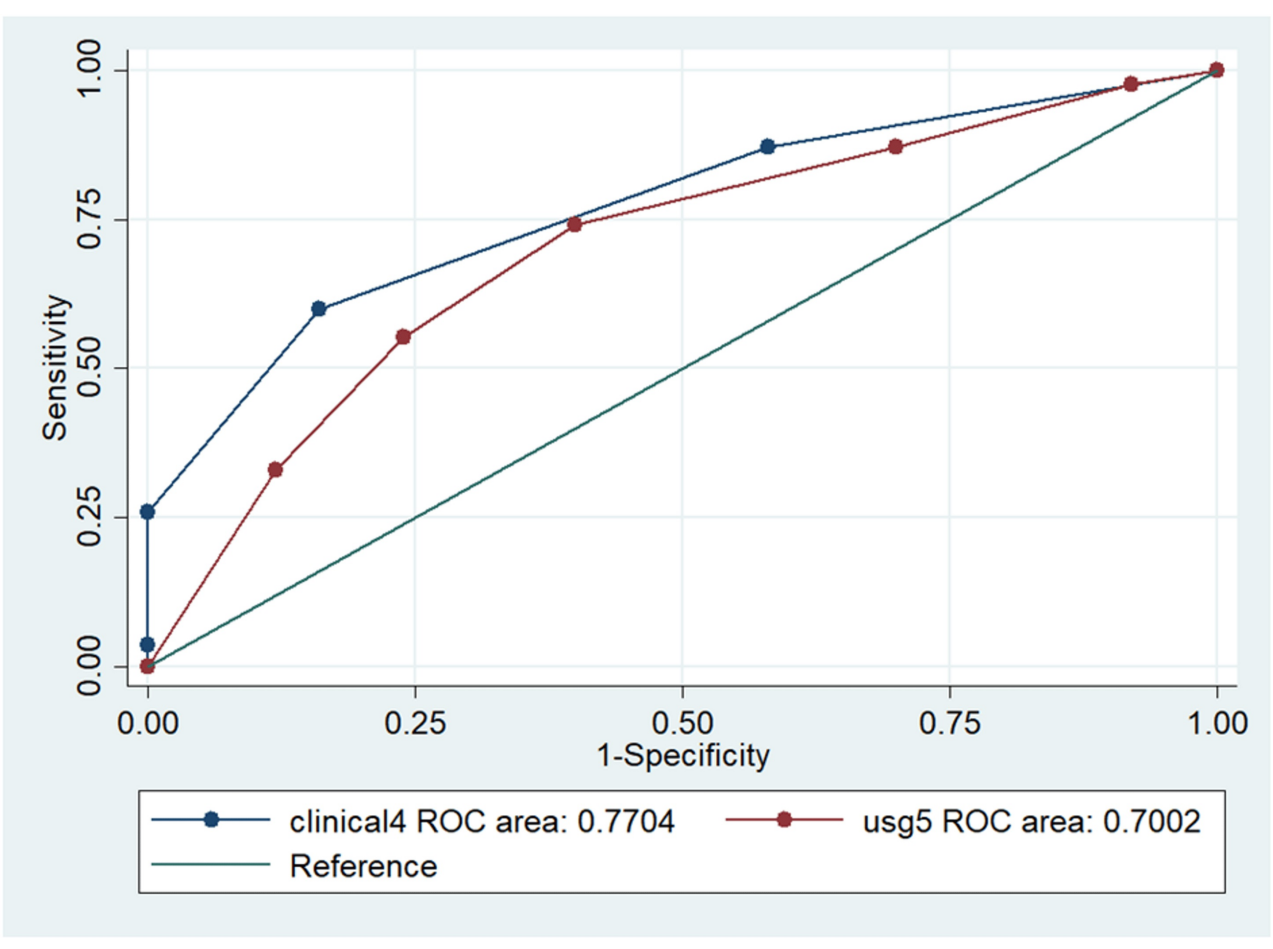
ROC curve comparison of significant clinical and significant ultrasound parameters. According to the ROC curves, the AUC for clinical parameters was determined to be 0.77 (95% CI: 0.69-0.84), while that of the ultrasound was 0.70 (95% CI: 0.61-0.79). ROC, receiver operating characteristic; AUC, area under the curve

ROC curves were constructed separately for the clinical and ultrasound parameters. The comparison between them is illustrated in Figure 4. According to the ROC curves, the AUC for clinical parameters was determined to be 0.77 (95% CI: 0.69-0.84), while that of the ultrasound was 0.70 (95% CI: 0.61-0.79). The two parameters did not exhibit any statistically significant differences.

## Discussion

The evaluation of airways and the capability to predict DL and difficulty in intubation have always been integral to the clinical assessment performed by an anesthesiologist. Since the inception of the Mallampati grading system in 1983 [8], several other parameters have been studied and remain the subject of ongoing research in the prediction of preoperative airway difficulty. Over a period, the individual indices were modified to form composite indices, such as the Wilson score [23] and Benumof score [5]. Despite this, the search for a conclusive method to evaluate difficult airways remains elusive, resulting in the absence of a definite predictor. It has been documented that there are approximately 600 deaths each year worldwide because of the inability to secure an airway. Karl Dussik introduced ultrasound in 1942, and its use in airway assessment became prominent during the early 2000s [24]. However, the current role of ultrasound in this context remains underdeveloped, lacking established parameters and definitive values.

Most of the studies [25-29] used Cormack-Lehane grades IIb, III, and IV as the preferred method for defining DL, while others employed the intubation difficulty scale [30,31]. Our description aligns with Koh et al.’s classification, categorizing DVL as CL grades IIb, III, and IV [3]. Our study revealed a 63% occurrence of a difficult airway, which is considerably higher compared to the reported prevalence of 8% to 10% in previous studies [10,13,32]. This is due to the age of 62 (46%) patients being 50-60 years, constituting the majority. A notable issue is that 44 (32.6%) out of 135 patients exhibited comorbidities, including type 2 diabetes, hypothyroidism, and obesity, which heightened the chance of DL. The occurrence of DL was evaluated in our study through the observation of the CL grade during direct laryngoscopy without employing the BURP (backward, upward, right side pressure) maneuver. Takahata et al. found that this method reduced the incidence of inability to view any part of the glottis by 5% [33].

Ultrasound parameters

Komatsu et al. were the first to investigate the potential of NTVC as an indicator for DL [34]. Their study concluded that it did not prove to be a reliable parameter in predicting difficulty in obese patients. On the other hand, Ezri et al. proposed it as a promising predictor of DL [27]. The findings of our study show that a thickness of 0.33 cm at NTVC does not serve as a reliable indicator for anticipating a difficult airway, consistent with the outcomes of Komatsu et al. [34].

Adhikari et al. used NTTM thickness assessment as a significant indicator for forecasting a DL. [12]. The results of our study show that a thickness of 0.67 cm at NTTM served as a reliable indicator for anticipating a difficult airway, consistent with the findings of Adhikari et al. Ezri et al. used the assessment of soft tissue thickness at the suprasternal notch as a reliable indicator for expecting a DL [27]. The results of our investigation indicate that a thickness of 1.01 cm at the NTSN level may reliably predict a DL, consistent with the findings of Ezri et al. [34].

Ezri et al. proposed that evaluating anterior soft tissue thickness at the hyoid level may be a significant indicator for predicting DL [27]. Given that the thickness at the hyoid level was significant just on the left side and not on the right, we concluded that this criterion may not be effective in predicting DL in the study we conducted. Gupta et al. used the measurement of PES thickness as a valuable indicator of a DL [13]. The results from our study provide evidence that a PES thickness of 0.57 cm was a reliable indicator for anticipating a DL, in line with the research conducted by Gupta et al. [13].

The research by Wojtczak indicates that evaluating the HMR may be a significant predictor of DL [14]. The HMR is not a reliable indicator for anticipating a DL in our study, in contrast to the findings of Wojtczak [14]. However, this study specifically examined HMD-N, which was determined to be a reliable indicator.

In their studies, Wojtczak [14] and Parameswari et al. [32] have highlighted the significance of measuring TV as a valuable parameter for expecting a DL. The study's results indicate that a TV of 72.15 cm3 serves as a valid predictor for DL, corroborating the findings of prior research by Wojtczak and Parameswari et al. [14,32].

Clinical parameters

This study identifies MMT, NC, TMH, and dental condition as significant clinical parameters. Carvalho et al. conducted a meta-analysis to evaluate the predictors of DL in adults and concluded that TMH exhibited considerable predictive accuracy in DLs conducted on adult patients from diverse populations, surpassing the performance of most previously evaluated bedside tests [35]. The studies by Rao et al. and Etezadi et al. suggested that TMH exhibits greater precision in predicting DL when compared to established anatomical measurements [10,19]. According to Riad et al. [36] and Hirmanpour et al. [37], NC was one of the independent indicators of DL, which supports the findings of the present study.

In this research, the sensitivity and specificity of the modified Mallampati grade for a grade of 3 or higher were found to be 57.65% and 78%, respectively. According to the findings of the research conducted by Yemam et al., the sensitivity of MMT in predicting DL was 46%, and its specificity was 93% [21]. According to the research carried out by Parameswari et al., MMT exhibited the highest levels of both sensitivity and specificity among the clinical parameters [32].

When all the significant clinical parameters were used together, they had 100% specificity. The specificity remains 100% even when three or more of the above parameters were used to predict DL. When all the five significant ultrasound parameters were used together, they had 100% specificity to predict DL. A follow-up research was conducted by Narkhede et al. to determine the predictors of difficult intubation in Indian patients [38]. Rather than focusing on a single component, they concluded that the most effective strategy to anticipate DL is to consider a combination of variables. This result lends credence to the conclusions of the current research.

Based on the findings of this study, we can infer that routine clinical airway examination and ultrasound examination yield comparable results in predicting DL. Therefore, the routine use of clinical airway examination still holds good in predicting DL.

Limitations

The majority of patients in our research were over the age of 50, which may have affected the findings due to the high occurrence of DL. The computation of the ultrasound parameters in this study was conducted by a single anesthesiologist. The cut-off values used for ultrasound parameters were not considered as gold standard values and were only calculated using ROC curves in a subset of the population studied.

## Conclusions

The ultrasound parameters that are significant are soft tissue thickness at the level of the thyrohyoid membrane, suprasternal notch, PES, distance from the hyoid to the mentum in neutral position, and TV. These might need more studies for validation and cut-off values. The clinical parameters that are significant are modified Mallampati grade, NC, TMH, and condition of teeth. Based on the findings, we observed that both clinical and ultrasound parameters are comparable to predict DL. Although ultrasound airway examination is an intriguing technique that allows visualization of deeper structures, our study shows it is not superior to clinical airway examination. This finding, combined with the steep learning curve required for ultrasound, suggests that clinical examination remains equally effective for predicting difficult laryngoscopy. Larger sample sizes with varying age, population, and ethnicity are needed to validate our results.
